# From wrist data to lifespan: elucidating inflammation-driven biological aging via activity rhythms captured by wearable devices

**DOI:** 10.1038/s41514-026-00349-x

**Published:** 2026-02-26

**Authors:** Jinjoo Shim, Faraz Bishehsari, Mahboobeh Mahdavinia, Jamie M. Zeitzer, Elgar Fleisch, Filipe Barata

**Affiliations:** 1 Centre for Digital Health Interventions, ETH Zurich, Zurich, Switzerland; 2 Department of Biostatistics, Harvard University, Boston, MA USA; 3 Gastroenterology Research Center (GRC), Department of Internal Medicine, University of Texas Health Science Center at Houston, Houston, TX USA; 4 MD Anderson Cancer Center-UTHealth Houston Graduate School of Biomedical Sciences, Houston, TX USA; 5 Division of Allergy and Immunology, Department of Internal Medicine, University of Texas Health Science Center at Houston, Houston, TX USA; 6 Department of Psychiatry and Behavioral Sciences, Stanford University, Stanford, CA USA; 7 Centre for Digital Health Interventions, University of St. Gallen, St. Gallen, Switzerland

**Keywords:** Biomarkers, Diseases, Neuroscience, Physiology

## Abstract

Systemic inflammation (“inflammaging”) accelerates biological aging and drives cardiovascular, metabolic, and neurodegenerative disease. Circadian rhythms regulate the amplitude and timing of immune responses, yet their mechanistic role in inflammation and longevity remains unexplored. In 62,000 adults with 7-day wearable accelerometry, interpretable machine learning model identified rhythm amplitude, stability, and moderate-to-vigorous physical activity (MVPA) as dominant predictors of accelerated aging. In a subset of 1521 participants (35% male) with available data on the systemic immune-inflammation index (SII), we further examined the associations between behavioral rhythmicity and inflammation. Low amplitude and poor rhythm stability were associated with 0.31 and 0.18 SD higher SII; low MVPA to 0.33 SD higher SII in men (all *p* < 0.05). Adding 15-min bout to daily MVPA or improving rhythm stability by 10–14% mitigated these effects. Sex-stratified mediation analysis revealed that inflammation accounted for 26% of the mortality risk associated with insufficient MVPA, 14% with rhythm irregularity, and 8% with low amplitude only in men. These findings position rest-activity rhythms as digital biomarkers linking daily rhythmicity to inflammation and survival and identify inflammation as a modifiable target for personalized interventions to foster healthy aging.

## Introduction

In recent years, chronic systemic inflammation has emerged as a critical pathological mechanism accelerating the aging process and increasing susceptibility to a wide spectrum of age-related diseases, including cardiovascular, metabolic, autoimmune, and neurodegenerative disorders^1–3^. The state of chronic, low-grade inflammation known as “inflammaging” is now regarded as a core, integrative hallmark of biological aging^3^. It arises from the interplay of cumulative macromolecular damage, age-related immune dysregulation, and modifiable environmental exposures. Epidemiological data indicate that elevated inflammatory tone is not an inevitable consequence of aging; instead, it reflects individual differences in lifestyle, developmental history, and other exogenous factors that shape inflammatory trajectories^4,5^. Accordingly, lifestyle-based interventions that attenuate inflammaging hold promise for reducing the burden of age-associated diseases.

Concurrently, circadian rhythms, nearly 24-h endogenous oscillations regulating essential physiological and behavioral processes like sleep-wake cycles, thermoregulation, hormonal secretion, and immune responses, are increasingly recognized as central to the aging process^6^. Disruptions in circadian rhythms accelerate aging and increase the risk of chronic diseases such as metabolic syndrome, cancer, and neurodegeneration^7,8^. Although in-lab monitoring in research facilities is the gold standard for assessing circadian rhythms, this approach is not feasible for free-living individuals and is impractical at the population level. Wearable devices offer continuous, passive, and non-invasive monitoring of rest-activity rhythmicity and physical activity from which the robustness of the circadian clock can be inferred^9^. These devices collect objective, quantifiable physiological and behavioral data (e.g., physical activity, heart rate, sleep patterns), referred to as “digital biomarkers”, that predict, associate, and evaluate health outcomes^9^. Previous research on digital biomarkers showed that robust amplitude of daily rest-activity rhythms, consistent sleep-wake cycles, and sufficient moderate-to-vigorous physical activity (MVPA) are linked to reduced risks of developing chronic diseases and mortality^10–12^. Building on this foundation, we recently introduced CosinorAge, a novel digital biomarker of aging and healthspan derived from wearable-based daily rest-activity rhythms. This biomarker has demonstrated predictive accuracy for the risk of mortality and other age-related outcomes^13^.

While these findings highlight the biological and prognostic relevance of rest-activity rhythms, the underlying pathways through which rest-activity rhythms influence biological aging, particularly through inflammation, remain unexplored. Existing analytical frameworks predominantly model distal endpoints such as disease onset and mortality, thereby overlooking inflammation as a proximate outcome. Yet systemic inflammation may serve as a modifiable, biologically salient marker that tracks dynamic physiological shifts and is amenable to short-term behavioral or therapeutic intervention.

To address this research gap, we aim to elucidate the potential pathway linking rest-activity rhythms, systemic inflammation, and biological aging. Our approach integrates wearable-derived activity data with a data-driven approach, consisting of three components. First, we extract multidimensional features of daily rest-activity rhythms, characterizing timing, intensity, stability, and periodicity, using continuous 7-day accelerometer recordings from a large prospective cohort. Second, we employ a data-driven machine learning framework to identify key contributing wearable features to predict accelerated vs decelerated CosinorAge from complex, high-dimensional data. CosinorAge, a rest-activity rhythm-based biological age measure derived from 7-day wrist accelerometry, integrates three parameters – MESOR (rhythm-adjusted mean activity level), amplitude (half the extent of the variation), and acrophase (timing of peak activity) – obtained from cosinor analysis into a Gompertz proportional hazards model to estimate 5-year mortality risk. Developed and externally validated in ~80,000 UK and US adults, CosinorAge offers a simple and interpretable framework for wearable-derived aging assessment. Building on this, we employed SHapley Additive exPlanation (SHAP) algorithm to identify the relative importance and directional contributions of key contributing wearable features. Third, we evaluate the associations between these key features on proximal (inflammation) outcome. We also examine the interaction effects between rest-activity rhythm amplitude, regularity, and physical activity intensity to determine whether one behavioral domain may compensate for or modulate the other. Lastly, we examined the role of systemic inflammation in mediating the effect of individuals’ rest-activity patterns and physical activity on aging-related mortality.

This study develops an explainable digital biomarker model for aging, highlights systemic inflammation as a potential mechanism, and underscores the translational value of wearable-derived biomarkers in health outcomes.

## Results

### Study population

The study population included 62,364 participants (40.0% male), with a median age of 58.0 years (IQR 51.0–63.0). Baseline demographic and wearable-derived characteristics were well balanced between the Training+Validation and Test sets (Table 1). Among participants, 61.0% were in paid employment at the time of study inclusion. A total of 41.1% were classified as overweight (BMI 25–29.9 kg/m^2^) and 18.0% as obese (BMI ≥ 30 kg/m^2^). The Townsend Deprivation Index (TDI), a census-based socioeconomic deprivation index, did not differ significantly between two groups.

**Table 1:** Baseline characteristics

Median (IQR) or *N* (%)	Training + Validation (*n* = 49,890)	Test (*n* = 12,474)	*p*-value
Age (years)	58.00 [51.00, 63.00]	58.00 [51.00, 63.00]	0.569
Sex			
Female	29,919 (60.0)	7497 (60.1)	0.797
Male	19,971 (40.0)	4977 (39.9)	
Race/Ethnicity			
White	48,773 (97.8)	12,156 (97.5)	0.042
Non-White	1117 (2.2)	318 (2.5)	
Townsend deprivation index	−2.51 [−3.85, −0.33]	−2.49 [−3.82, −0.24]	0.147
BMI			
Normal or underweight	20,626 (41.3)	5203 (41.7)	0.617
Overweight	20,534 (41.2)	5074 (40.7)	
Obese	8730 (17.5)	2197 (17.6)	
Employment status			
Paid employment	30,564 (61.3)	7550 (60.5)	0.134
Unpaid	19,326 (38.7)	4924 (39.5)	
Education			
College or above	22,115 (44.3)	5541 (44.4)	0.933
High school or equivalent	23,835 (47.8)	5939 (47.6)	
Less than high school	3940 (7.9)	994 (8.0)	
Smoking status			
Never	29,105 (58.3)	7298 (58.5)	0.938
Previous	17,712 (35.5)	4414 (35.4)	
Current	3073 (6.2)	762 (6.1)	
Alcohol consumption			
Not current	2634 (5.3)	671 (5.4)	0.56
<3 times/week	22,483 (45.1)	5675 (45.5)	
≥3 times/week	24,773 (49.7)	6128 (49.1)	
Employment status			
Paid employment	30,564 (61.3)	7550 (60.5)	0.134
Unpaid	19,326 (38.7)	4924 (39.5)	
History of shift work			
Yes	46,308 (92.8)	11,578 (92.8)	1
No	3582 (7.2)	896 (7.2)	
Comorbidities			
Hypertension	11,654 (23.4)	2864 (23.0)	0.351
Diabetes	1680 (3.4)	416 (3.3)	0.879
Cardiovascular disease	160 (0.3)	33 (0.3)	0.358
Cancer	7458 (14.9)	1849 (14.8)	0.734
Neurodegenerative disease	83 (0.2)	20 (0.2)	0.98
Chronic respiratory disease	661 (1.3)	170 (1.4)	0.774
Wearable-derived features			
Cosinor amplitude, mg	43.16 [34.16, 56.28]	43.12 [34.15, 56.09]	0.846
Cosinor amplitude variability	59.99 [28.98, 91.10]	59.41 [28.03, 89.59]	0.020
Relative amplitude	0.89 [0.87, 0.91]	0.89 [0.87, 0.91]	0.178
Relative amplitude variability	3.64 [2.53, 5.31]	3.64 [2.53, 5.37]	0.396
Acrophase, clock hour	13.93 [13.19, 14.69]	13.93 [13.20, 14.70]	0.651
Acrophase variability	9.28 [6.42, 12.93]	9.17 [6.32, 12.93]	0.097
Up-mesor, clock hour	7.77 [7.08, 8.55]	7.77 [7.07, 8.55]	0.707
Up-mesor variability	20.14 [12.19, 28.86]	19.82 [12.08, 28.57]	0.154
Interdaily stability	0.34 [0.30, 0.38]	0.34 [0.30, 0.38]	0.311
Pseudo-F	120.81 [60.64, 197.64]	122.60 [60.88, 200.35]	0.085
MVPA, hours/day	0.58 [0.30, 1.00]	0.59 [0.30, 1.00]	0.794
Insufficient MVPA (<150 MVPA minutes/week)	24,746 (49.6)	6129 (49.1)	0.356
MVPA variability	91.15 [63.45, 127.68]	90.72 [62.90, 127.89]	0.963
Sedentary behavior, hours/day	9.28 [8.09, 10.45]	9.27 [8.07, 10.43]	0.324
Sedentary behavior variability	16.89 [12.25, 22.74]	16.84 [12.17, 22.67]	0.652
Intensity gradient	−2.56 [−2.67, −2.45]	−2.56 [−2.67, −2.46]	0.081
PAEE	46.00 [43.59, 48.54]	45.99 [43.62, 48.51]	0.597
Sleep onset, clock hour	27.29 [26.69, 27.92]	27.28 [26.67, 27.92]	0.458
Sleep efficiency, %	0.77 [0.73, 0.81]	0.77 [0.73, 0.81]	0.540
Sleep duration, hours/day	7.37 [6.83, 7.88]	7.38 [6.83, 7.89]	0.486
Sleep duration variability	0.77 [0.58, 1.01]	0.77 [0.59, 1.02]	0.244
DFA1	1.02 [0.98, 1.06]	1.02 [0.98, 1.06]	0.775
DFA2	0.94 [0.88, 1.01]	0.94 [0.88, 1.01]	0.149
fPC2 (Activity initiation)	−5.20 [−118.73, 109.55]	−6.18 [−118.80, 107.58]	0.535
fPC3 (Timing of peak activity)	5.78 [−98.12, 109.97]	8.02 [−100.49, 111.03]	0.502
fPC4 (Unimodal vs. bimodal activity)	1.78 [−82.24, 82.08]	0.84 [−84.61, 81.70]	0.556

The *p*-values are computed using Wilcoxon rank-sum test for continuous variables and chi-square test for categorical variables.

*BMI* body mass index, *MVPA* moderate-to-vigorous physical activity, *PAEE* physical activity energy expenditure, *DFA1* scaling exponent from detrended fluctuation analysis using shorter-time scale (<2 h), *DFA2* scaling exponent from detrended fluctuation analysis using longer-time scale (>2 h); PC2, PC3, PC4, 2nd, 3rd, 4th principal component from functional principal component analysis.

Wearable-derived measures indicated robust daily rest-activity rhythmicity, with a median cosinor amplitude of 43.1 mg (IQR 34.2–56.3) and a relative amplitude of 0.89 (IQR 0.87–0.91). The median acrophase (peak activity timing) occurred at 13.93 h (IQR 13.19–14.69), while the up-mesor (rest-to-active transition timing) was 7.77 h (IQR 7.08–8.55). Physical activity patterns showed a median moderate-to-vigorous physical activity (MVPA) duration of 0.58 h/day (IQR 0.30–1.00), sedentary behavior of 9.28 h/day (IQR 8.09–10.45), and total sleep duration of 7.37 h/day (IQR 6.83–7.88). 49.5% of participants were classified as having insufficient MVPA defined as engaging in less than 150 min per week of moderate-to-vigorous physical activity. Sleep efficiency had a median value of 0.77 (IQR 0.73–0.81), indicating stable sleep quality across groups. Interdaily stability, reflecting the regularity of daily rhythms, had a median of 0.34 (IQR 0.30–0.38).

Functional principal component analysis (fPCA) identified four orthogonal rhythmic components. Because fPC1 was highly correlated with cosinor amplitude, it was excluded from subsequent analyses. The remaining components (fPC2–fPC4) captured distinct temporal aspects of rest-activity rhythms: fPC2 corresponded to the timing of activity initiation, fPC3 to the timing of peak activity, and fPC4 to unimodal versus bimodal activity patterns. Consistent with findings from previous studies^14^, these behavioral rest-activity rhythm characteristics present volume, timing, structure, and regularity dimensions of daily activity patterns. Temporal complexity measures from detrended fluctuation analysis showed median scaling exponents of 1.02 (DFA1) and 0.94 (DFA2), indicating preserved short- and long-range autocorrelations.

To assess day-to-day behavioral consistency, we further analyzed daily-level metrics of rest-activity rhythmicity, physical activity, quantifying between-day fluctuations using the coefficient of variation, defined as the ratio of the standard deviation to the mean. The greatest variability was observed for MVPA duration (91%), cosinor amplitude (59%), up-mesor timing (20%), and sedentary duration (17%).

### SHAP analysis and influential wearable features

**Fig. 1:**
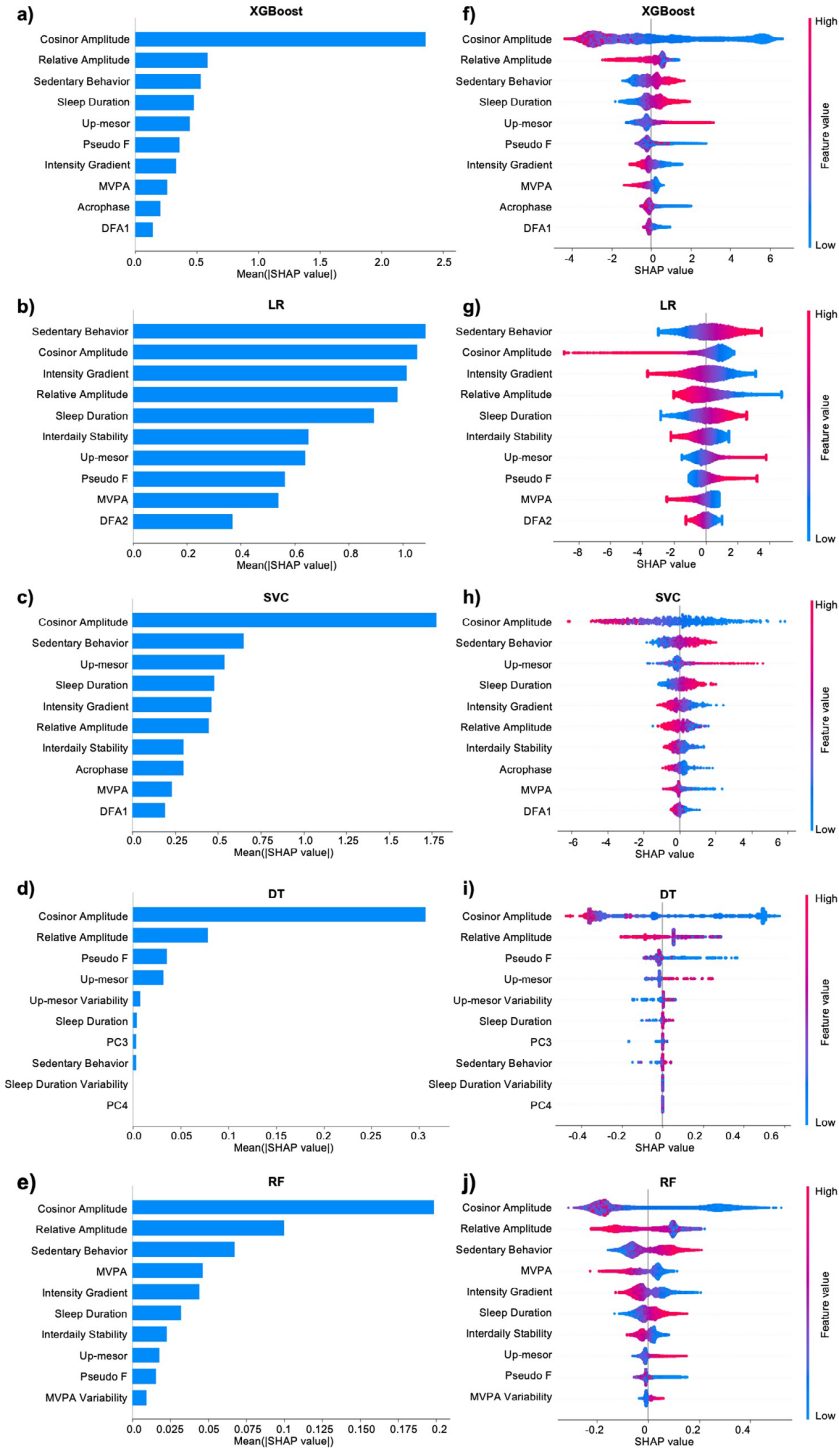
SHAP analysis for machine learning models estimating biological age. **a**–**e** SHAP importance plots ranking of the top 10 influential features based on mean absolute SHAP values. Features are sorted according to global contribution. **f**–**j** SHAP summary plots of the impact of individual features on model output. Red dots represent higher feature values, while blue dots denote lower feature values. Positive SHAP values represent the contribution of the feature to a higher biological age prediction, while negative SHAP values represent the contribution of the feature to a lower biological age prediction. For example, lower cosinor amplitude, lower relative amplitude, and higher sedentary behavior contribute to higher biological age. XGBoost extreme gradient boosting, LR logistic regression, SVC support vector classifier, DT decision tree, RF random forest, MVPA moderate-to-vigorous physical activity, DFA1 scaling exponent from detrended fluctuation analysis using shorter-time scale (<2 h), DFA2 scaling exponent from detrended fluctuation analysis using longer-time scale (>2 h), PC2, PC3, PC4, 2nd, 3rd, 4th principal component from functional principal component analysis.

The dataset comprises 52.2% of individuals classified as having higher estimated biological age than chronological age and 47.8% classified as having lower estimated biological age. Among the machine learning (ML) models evaluated, extreme gradient boosting (XGBoost) exhibits the best performance, achieving an area under the curve (AUC) of 0.971, an accuracy of 0.903, and an Mattews correlation coefficient of 0.805 (Supplementary Table 4). The top 10 features contributing to model predictions from each ML model are shown in SHAP importance plots (Fig. 1a–e). These wearable features characterize behavioral rest-activity patterns and physical activity metrics that are associated with biological aging estimates.

The SHAP summary plots (Fig. 1f–j) further provide insights into the directional contributions of the top features to the model’s predictions. In these plots, red indicates higher feature values, while blue corresponds to lower feature values. Positive SHAP values represent the contribution of the feature to higher biological age estimate, while negative SHAP values represent the contribution of the feature to lower biological age estimate. Across all ML models, low cosinor amplitude, low relative amplitude, and prolonged sedentary behavior consistently appear among the features most strongly associated with higher predicted biological age. Goodness of fit measure, such as the pseudo-F statistic and rhythm regularity metric, such as interdaily stability, also rank highly among the top predictors. Lower values of these metrics correlate with higher SHAP values, suggesting that reduced rhythm stability contributes to a higher biological age estimate. Additionally, features related to activity timing, including up-mesor (the timing of activity initiation) and acrophase (the timing of peak activity), contribute valuable information. Intensity gradient and MVPA exhibit that increased levels of these variables are associated with lower biological age. In summary, rest-activity rhythms and physical activity domains prominently feature in the list of top features, while sleep metrics beyond sleep duration and the data-adaptive approach are less represented.

### Association between wearable features and proximal inflammation

In a subset of 1521 participants (35% male) with available data on the systemic immune-inflammation index (SII), we evaluated how key wearable features are related to proximal inflammation, identifying plausible pathways of wearable-derived digital biomarkers to aging processes. Specifically, we assessed SII levels within six months of wearable data collection as a proximal inflammation among individuals with complete laboratory tests for complete blood count. SII has recently been recognized as a prognostic indicator for various cancers^15^, coronary artery disease^16^, and inflammatory diseases^17^. Furthermore, SII has shown significant associations with mortality^18^ and phenotypic age^19^, demonstrating its potential as a marker indicative of the aging process.

Three wearable-derived features, namely relative amplitude (RA), interdaily stability (IS), and MVPA duration, capture distinct yet related aspects of rest-activity rhythms and physical activity. The pairwise Spearman correlation coefficients among these features ranged from 0.11 (MVPA–IS), 0.29 (IS–RA), and 0.37 (MVPA–RA), indicating weak to moderate correlations.

In multivariable generalized linear models controlling for demographics, socioeconomic, and comorbidities, all three features show significant associations with systemic inflammation levels (Table 2). Individuals in the lowest quartile of relative amplitude exhibit substantially higher SII levels by 0.31 SD (95% CI: 0.14–0.48) compared to those in the highest quartile (*p* = 0.0005). In a trend analysis of relative amplitude quartiles, each one-quartile decrease in relative amplitude (reflecting decreased rhythm amplitude) is associated with a 0.11 SD increase in SII, suggesting the importance of wearable-derived rhythm amplitude on inflammatory marker level (95% CI: 0.05–0.16; *p* = 0.0001).

**Table 2:** Associations between wearable features and systemic inflammation

Main effects		
Feature	Beta (95% CI)	*p*-value
Relative amplitude (continuous)^a^	0.11 (0.05, 0.16)	0.0001*^b^
Relative amplitude (quartiles)		
Q4 (high amplitude)	Reference	–
Q3	0.03 (−0.14, 0.20)	0.74
Q2	0.17 (−0.003, 0.34)	0.05
Q1 (low amplitude)	0.31 (0.14, 0.48)	0.0005*^b^
Interdaily stability (continuous)^a^	0.06 (0.004, 0.11)	0.035*
Interdaily stability (quartiles)		
Q4 (good regularity)	Reference	–
Q3	0.13 (−0.02, 0.28)	0.094
Q2	0.16 (0.002, 0.32)	0.048*
Q1 (irregularity)	0.18 (0.004, 0.35)	0.045*
MVPA (categorical)		
Male: insufficient vs. sufficient	0.33 (0.006, 0.64)	0.046*
Female: insufficient vs. sufficient	−0.002 (−0.10, 0.10)	0.969
Interaction effects		
Feature	Beta (95% CI)	*p*-value
Amplitude & regularity		
High amplitude & good regularity	Reference	–
High amplitude & irregularity	0.24 (−0.29, 0.77)	0.384
Low amplitude & good regularity	0.03 (−0.47, 0.52)	0.918
Low amplitude & irregularity	0.43 (0.05, 0.83)	0.038*
MVPA & regularity		
Sufficient MVPA & good regularity	Reference	–
Sufficient MVPA & irregularity	0.001 (−0.26, 0.26)	0.992
Insufficient MVPA & good regularity	0.06 (−0.18, 0.30)	0.651
Insufficient MVPA & irregularity	0.25 (0.02, 0.47)	0.033*
Amplitude & MVPA		
High amplitude & sufficient MVPA	Reference	–
High amplitude & insufficient MVPA	0.15 (−0.20, 0.50)	0.400
Low amplitude & sufficient MVPA	−0.02 (−0.35, 0.32)	0.926
Low amplitude & insufficient MVPA	0.28 (0.05, 0.51)	0.018*

Relative amplitude and interdaily stability were analyzed both as quartile groups (from high amplitude/good regularity to low amplitude/irregularity) and as continuous variables representing the numeric trend across these quartiles. For the main effects, generalized linear models were adjusted for age, sex, ethnicity/race, TDI, BMI, employment status, shift work, smoking status, alcohol consumption, sleep duration, and comorbidities (hypertension, diabetes, cardiovascular disease, cancer, neurodegenerative disease, and chronic respiratory diseases). For relative amplitude and interdaily stability, models were additionally adjusted for MVPA duration. For MVPA, models were additionally adjusted for interdaily stability. Interaction effect models were adjusted for age, sex, ethnicity/race, TDI, BMI, employment status, and shirt work for convergence. The outcome variable was Z-scored, and regression results are expressed per one-standard deviation (1 SD) increase in the systemic immune inflammation index (SII).

^a^The positive coefficient reflects a quartile decrease (i.e., decreased rhythm amplitude and decreased rhythm regularity) is associated with higher systemic inflammation.

^b^Indicates *p*-value remained significant after adjusting for multiple comparisons using the false. discovery rate procedure by Benjamini and Hochberg.

*p values < 0.05.

A similar association is observed with interdaily stability, a measure of day-to-day rhythm stability. In fully adjusted generalized linear models, individuals in the lowest quartile of interdaily stability, indicative of poor day-to-day rhythm stability or high irregularity, have SII levels that were 0.18 SD higher than those in the highest quartile, representing stable day-to-day rhythmicity (95% CI: 0.004–0.35, *p* = 0.045). Trend analysis reveals that each quartile decrease in interdaily rhythmicity (reflecting increased irregularity) corresponds to a 0.06 SD increase in SII (95% CI: 0.004–0.11, *p* = 0.035), indicating that both weaker and less stable behavioral rhythms are associated with heightened inflammation.

Furthermore, we observe a sex-specific association between physical activity and SII levels. In males, insufficient MVPA duration is associated with a significant increase in inflammation, with SII levels rising by 0.33 SD (95% CI: 0.006–0.64, *p* = 0.046). However, this association is not statistically significant in females in study cohort.

Sensitivity analyses excluding participants reporting shift work or sleep disturbances yielded consistent results (Supplementary Tables 7, 8).

### Joint effect of rest-activity rhythms and physical activity on systemic inflammation

To assess how rest-activity rhythm amplitude, rhythm regularity, and physical activity jointly influence systemic inflammation, we analyzed interaction effects across these behavioral domains (Table 2). Interaction effects were examined using generalized linear models adjusted for age, sex, ethnicity/race, TDI, BMI, employment status, and shift work. The strongest association is observed in participants with both insufficient MVPA and poor rhythm regularity, who exhibit a 0.25 SD increase in SII (95% CI: 0.02–0.47, *p* = 0.033) compared to the reference group with sufficient MVPA and good regularity. In contrast, individuals with only one adverse factor, insufficient MVPA but good regularity (β = 0.06; 95% CI: −0.18–0.30; *p* = 0.651) or sufficient MVPA but irregular rhythms (β = 0.001; 95% CI: −0.26–0.264; *p* = 0.992), show modest, non-significant elevations in SII levels.

A similar trend is found for the interaction between rest-activity rhythm amplitude and regularity. Participants with both low amplitude and irregular rhythms exhibit a 0.43 SD increase in SII (95% CI: 0.05–0.83; *p* = 0.038) relative to those with high amplitude and good regularity. Individuals with one disrupted trait experience smaller, non-significant increases in SII (High Amplitude & Irregularity: β = 0.24, *p* = 0.384; Low Amplitude & Good Regularity: β = 0.03, *p* = 0.918).

Lastly, interaction between amplitude and MVPA reveals a significant elevation in SII among participants with both low amplitude and insufficient MVPA (β = 0.28; 95% CI: 0.05–0.51; *p* = 0.018), further emphasizing the additive burden of rest-activity rhythm disruption and activity-related impairments. In contrast, individuals with low amplitude but sufficient MVPA showed nearly no difference in SII (β = −0.02; 95% CI: −0.35–0.32; *p* = 0.926), suggesting that physical activity may offset the deleterious effects of dampened rhythms.

These findings together suggest that concurrent patterns of rest-activity rhythm irregularity and low physical activity are jointly associated with higher systemic inflammation. In particular, the combination of irregular rhythms and insufficient MVPA were associated with the greatest effect on inflammatory burden. Conversely, the results demonstrate the potential benefits of maintaining at least one healthy behavioral domain, as doing so may be associated with lower levels of systemic inflammation and greater physiological resilience.

### Reciprocal compensation between rhythm regularity and physical activity in biological aging

**Fig. 2:**
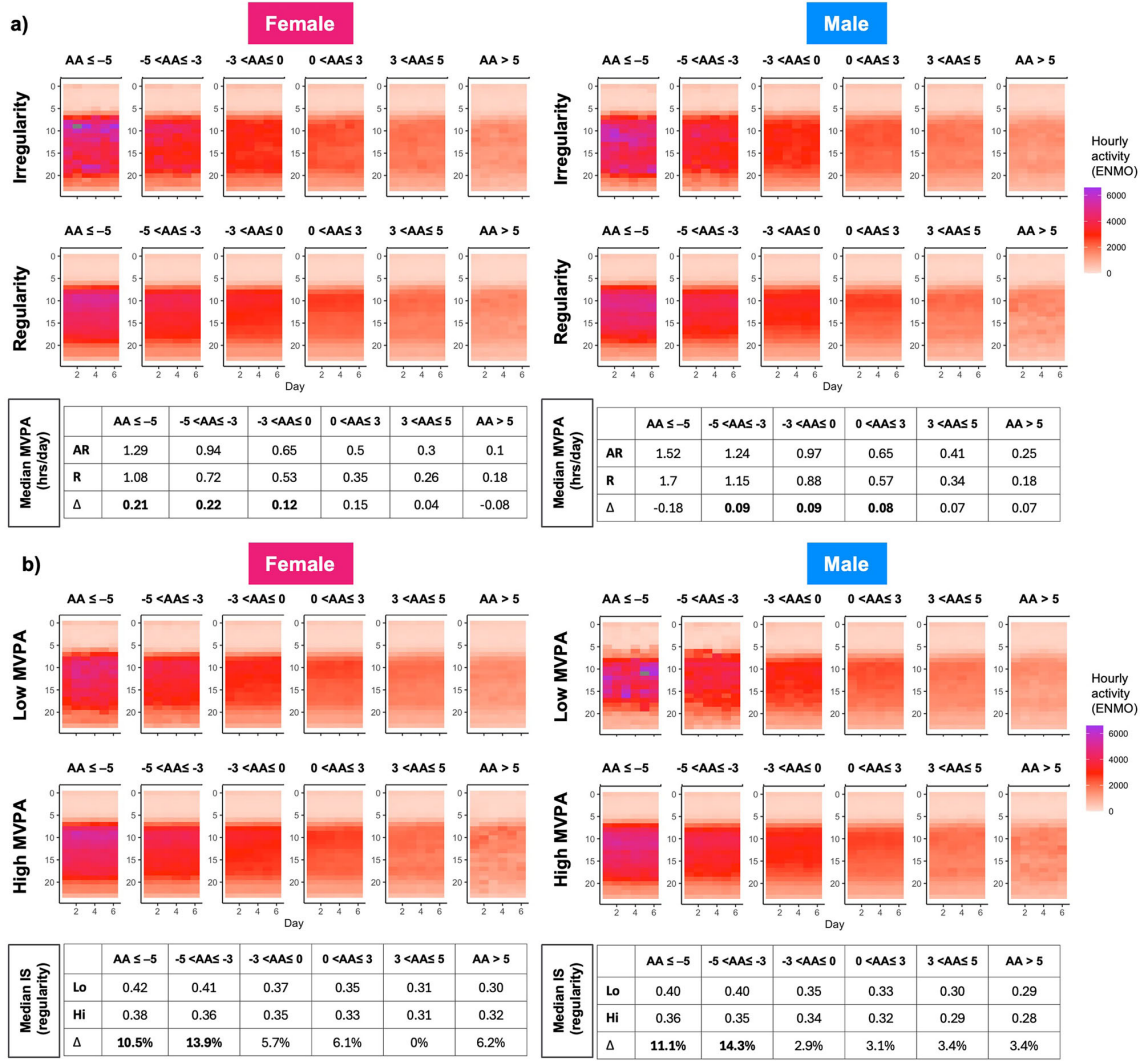
Sex-stratified heatmap analysis revealing reciprocal compensation between rhythm regularity and physical activity in relation to biological age acceleration. **a** Hourly physical activity levels visualized as heatmaps, stratified by rest-activity rhythm phenotype (irregular vs. regular) and biological aging rate, separately for females (left) and males (right). **b** Hourly physical activity levels visualized as heatmaps, stratified by MVPA phenotype (low vs. high MVPA) and biological aging rate, separately for females (left) and males (right). Each cell in the heatmaps represents average hourly activity, quantified using Euclidean Norm Minus One (ENMO) units derived from wrist-worn accelerometers. Aging acceleration is defined as the difference between estimated biological age (BA) and chronological age (CA), where age acceleration (AA) = BA – CA. Participants are grouped into age acceleration strata: (AA ≤ –5, –5 < AA ≤ –3, –3 < AA ≤ 0, 0 < AA ≤ 3, 3 < AA ≤ 5, AA > 5. Darker colors reflect higher physical activity levels. Median MVPA (hours/day) is reported for each rhythm phenotype subgroup in (**a**), while median interdaily stability (IS), a nonparametric measure of rhythm regularity (range 0–1), is reported for each MVPA subgroup in (**b**). In (**a**), AR and R denote “irregular rest-activity rhythm” and “regular rest-activity rhythm” groups, respectively. In (**b**), Lo and Hi denote “low MVPA” and “high MVPA” groups, respectively. Δ (%) in panel (**b**) refers to the relative difference in IS between fast and slow aging groups.

In this subset of 1521 participants, we examined how rest-activity rhythm regularity and physical activity jointly relate to biological aging by analyzing hourly activity heatmaps stratified by aging speed, sex, and behavioral phenotype (Fig. 2). Biological age acceleration was defined as difference between estimated biological age and chronological age (AA = BA-CA). Participants were grouped into age acceleration strata: AA ≤ –5, –5 < AA ≤ –3, –3 < AA ≤ 0, 0 < AA ≤ 3, 3 < AA ≤ 5, AA > 5.

Among individuals with biological age younger than their chronological age, those with irregular rest-activity rhythms exhibit higher levels of physical activity compared to their counterparts with regular rest-activity rhythms (Fig. 2a). For instance, in females whose estimated biological age is younger than chronological age by 5 or more years, the median MVPA duration is 1.29 h per day among those with irregular rhythms, compared to 1.08 h per day among those with regular rhythms, yielding a difference of ~12.6 min. A similar pattern is observed in females with biological age younger by 3–5 years, where the median MVPA is 0.94 h per day in the irregular group and 0.72 h in the regular group, corresponding to a difference of ~13.2 min.

This trend is attenuated in males. In the group with biological age younger than chronological age by 3–5 years, the median MVPA is 1.24 h per day for those with irregular rhythms and 1.15 h for those with regular rhythms (a difference of ~5.4 min). In the group with biological age is younger by 0–3 years, median MVPA is 0.97 h per day for irregular rhythm individuals and 0.88 h per day for those with regular rhythms. Across all rhythm regularity strata, both sexes exhibit a progressive decline in median MVPA with increasing biological age acceleration.

In parallel, analysis of rest-activity rhythm stability in individuals with low MVPA reveals an inverse but complementary trend (Fig. 2b). In females with low MVPA, median interdaily stability (IS) increases from 0.30 in the group with highest biological age acceleration (AA > 5) to 0.42 in the slowest aging group (AA ≤ −5). In males, IS similarly increases from 0.29 to 0.40.

The largest gain in rhythm stability is observed among individuals whose biological age is younger by 3–5 years. In this group, males exhibit an IS increase from 0.35 to 0.40, representing a +14.3% improvement, while females show an increase from 0.36 to 0.41, equivalent to a +13.9% change.

These findings reveal two reciprocal behavioral patterns: (1) among individuals with irregular rest-activity rhythms, MVPA is elevated in the context of younger biological age; and (2) among those with low MVPA, rest-activity rhythm regularity is enhanced in those with younger biological age. Together, these results suggest potential compensatory mechanisms between behavioral domains that may support healthier aging trajectories.

### Mediation analysis

In the full analytic cohort (*n* = 62,364), we observed 2277 deaths (3.6%) over a median follow-up of 8.09 years (IQR 7.5–8.6 years, comprising 1014 deaths in females (2.7%) and 1263 deaths in males (5.1%). We observed that low rest-activity rhythm amplitude and insufficient MVPA were significantly associated with higher all-cause mortality independent of demographic, socioeconomic, and clinical covariates (Supplementary Table 9). Building on these associations, we conducted a sex-stratified mediation analysis in the subcohort (*n* = 1521) to evaluate the role of systemic inflammation in mediating the relationship between wearable-derived digital biomarkers and distal aging outcome (Table 3). Within the subcohort with SII data available, 75 deaths occurred during a median follow-up of 7.98 years (IQR 7.5–8.4), corresponding to an overall mortality rate of 4.9% (3.8% in females [38 deaths] and 7.0% in males [37 deaths]). The majority of deaths were due to age-related chronic diseases, such as cancer (51%), cardiovascular disease (24%), and neurodegenerative disorders (7%) with only a small proportion attributable to external causes or infections (2%). These findings indicate that mortality in this age range primarily reflects distal aging outcomes rather than non-aging causes.

**Table 3:** Causal mediation analysis of systemic inflammation on the associations between wearable digital biomarkers and all-cause mortality

HR (95% CI)	Male	Female
Rhythm irregularity		
Natural indirect effect	1.11 (0.78, 1.56)	1.00 (0.66, 1.50)
Natural direct effect	1.68 (1.28, 2.21)	1.16 (0.84, 1.59)
Total effect	1.89 (1.57, 2.23)	1.15 (0.91, 1.45)
Mediated proportion^a^	14%	1%
Insufficient MVPA		
Natural indirect effect	1.07 (0.79, 1.45)	0.99 (0.81, 1.38)
Natural direct effect	1.18 (0.89, 1.62)	1.47 (1.09, 2.01)
Total effect	1.28 (1.16, 1.42)	1.45 (1.18, 1.82)
Mediated proportion^a^	26%	4%
Low amplitude		
Natural indirect effect	1.08 (0.72, 1.65)	1.02 (0.70, 1.41)
Natural direct effect	2.29 (1.48, 3.34)	1.34 (1.05, 1.73)
Total effect	2.47 (2.27, 2.69)	1.37 (1.12, 1.63)
Mediated proportion^a^	8%	9%

Mediation models are adjusted for age, TDI, BMI, employment status, and shift work. 95% CI are derived 1000 bootstrap sampling.

*HR* hazard ratio, *CI* confidence intervals.

^a^Proportion mediated = natural indeirect log-HR/total effect log-HR^58^.

In males, systemic inflammation mediates 8–26% of mortality risk, with the strongest mediation observed for insufficient MVPA followed by rest-activity rhythm irregularity and low amplitude (Table 3). For rhythm irregularity, both the total effect (HR 1.89, 95% CI 1.57–2.23) and the natural direct effect (HR 1.68, 95% CI 1.28–2.21) were significant, while the natural indirect effect through systemic inflammation (HR 1.11, 95% CI 0.78–1.56) accounted for 14% of the total association. For insufficient MVPA, the total hazard of mortality (HR 1.28, 95% CI 1.16–1.42) was partly explained by systemic inflammation (HR 1.07, 95% CI 0.79–1.45), corresponding to 26% mediation, suggesting that inflammation substantially contributes to mortality among males with low activity levels. For low amplitude, both the total (HR 2.47, 95% CI 2.27–2.69) and direct effects (HR 2.29, 95% CI 1.48–3.34) were strong, while the indirect effect via inflammation (HR 1.08, 95% CI 0.72–1.65) explained 8% of the total effect.

In females, both direct and indirect effects were generally smaller and less consistent than males. For rhythm irregularity, the total effect was insignificant (HR 1.15, 95% CI 0.91–1.45), with negligible mediation (HR 1.00, 95% CI 0.66–1.50; 1% of total). For insufficient MVPA, the total effect (HR 1.45, 95% CI 1.18–1.82) was driven primarily by the direct pathway (HR 1.47, 95% CI 1.09–2.01), while the indirect effect via inflammation (HR 0.99, 95% CI 0.81–1.38) accounted for 4% of the total association. For low amplitude, both the total (HR 1.37, 95% CI 1.12–1.63) and direct effects (HR 1.34, 95% CI 1.05–1.73) were significant, and the indirect effect (HR 1.02, 95% CI 0.70–1.41) mediated approximately 9% of the association. Collectively, these results show that systemic immune-inflammation index (SII), a measure of systemic inflammation accounted for higher proportion of mortality risk in males (8–26%) compared to females (1–9%), suggesting that systemic inflammation may contribute to the increased mortality associated with rest-activity disruption, although this effect appears to vary by sex. However, none of the natural indirect effects reached statistical significance.

## Discussion

In this study, we analyzed 7-day, 24-h wearable recordings from 62,000 adults to identify key behavioral predictors of accelerated biological aging using interpretable machine learning. In a subset of 1521 participants with available data on systemic inflammation, we further evaluated systemic inflammation as a potential pathway linking rest-activity rhythms to mortality risk. Our key finding is that wearable-derived rest-activity rhythms have the potential to serve as digital biomarkers for mortality risk, possibly through pathways such as systemic inflammation.

Our study, which examines population-level associations between wearable-derived rest-activity rhythm measures and aging trajectories, builds on strong preclinical evidence^20,21^ and advances current methodology in several important ways. First, our data-driven approach identified rest-activity rhythm amplitude, stability, timing, and intensity as distinct components uniquely related to aging. In addition to rhythm amplitude and sedentary duration, we showed that measures such as interdaily stability, pseudo-F statistic, and timing parameters provided additional discriminatory value for predicting age acceleration. These findings underscore that aging-related signals are shaped not only by overall activity intensity but also by the structure and regularity of daily rhythmicity. This aligns with growing evidence that reduced rhythm strength and lower stability are associated with several age-related outcomes including increased risks of frailty, poorer cognitive performance^22,23^, and elevated chronic disease incidence and mortality^11^ in midlife and older adulthood. Furthermore, a cross-sectional study of 52 healthy young adults showed that lower rhythm regularity and greater rhythm fragmentation were significantly associated with higher C-reactive protein and total cholesterol, and rest-activity rhythm regularity may emerge early in adulthood and be related to cardiometabolic health^24^.

Another distinctive contribution lies in how rest-activity rhythm features relate to systemic inflammation, a proximal marker of aging. Lower amplitude, irregular rhythms, and insufficient MVPA were associated with elevated inflammation, and counterfactual mediation analysis revealed that systemic inflammation accounts for a proportion of the mortality risk, particularly in males (up to 26%) than in females (up to 9%). This sex difference aligns with established sex-based immunological differences, which arise from both genetic and environmental influences^25,26^. Supporting this, a study of 60 adults reported that males exhibit a less favorable redox and inflammatory status than females, potentially reflecting reduced protection against the adverse effect of low activity^27^. In line with these studies, our work presents systemic inflammation as one possible pathway linking disruption in rest-activity rhythms to mortality, while additional mechanisms, such as autonomic imbalance, impaired sleep physiology, and metabolic dysregulation, likely contribute to the overall risk. Additionally, female undergo a shift in aging trajectory post menopause and generally live longer, both of which may challenge our ability to study proximal inflammation as a mediating mechanism in long-term aging here, given that our population age range was limited to 40–70.

Our joint analyses of rest-activity rhythm stability and physical activity introduce a new insight: these behavioral domains may compensate for one another in reducing inflammatory burden. Higher physical activity levels appeared to offset the adverse effect of low rhythm stability, whereas more stable daily rhythms mitigated the impact of insufficient physical activity on systemic inflammation. This compensatory pattern suggests that behavioral interventions may target the domain of greatest vulnerability. Such flexibility is particularly relevant for populations with structural constraints, such as shift workers or individuals with irregular schedules. These findings also carry broader public health implications, as irregular sleep timing, nighttime activity, social jetlag, and screen-driven rhythm fragmentation are widespread in young adults and tend to worsen with age-related physiological changes.

In this aspect, recent developments in digital devices, including wearables and smartwatches, expand the translational relevance of this work. Unlike traditional circadian assessments that rely on melatonin profiling and require invasive and costly biological sampling within a controlled environment, wearables offer continuous, non-invasive, and scalable monitoring of rest-activity cycles, sleep-wake timing, and physical activity in real-world settings^28^. Both research-grade actigraphy and consumer wearables have demonstrated validity for characterizing disruption in rest-activity rhythmicity, supporting their application in large-scale behavioral digital phenotyping^29–31^. When analyzed longitudinally, these data can be transformed into digital biomarkers that can track dynamic variations in rest-activity rhythm patterns and correlate, associate, and predict health outcomes and aging trajectories^9^. With digital devices becoming increasingly widespread and scalable, digital biomarkers offer a potential avenue for population-level surveillance and for delivering targeted behavioral guidance that may help mitigate inflammation-related aging risk.

This study has several strengths. First, the large sample size and temporal proximity between wearable measurements and inflammation assessments enhance the robustness of our findings. In contrast to the UK Biobank’s periodic assessment center visits, which may be years apart from accelerometer recordings, we incorporated primary care data that allows for more contemporaneous assessment of inflammatory status and improves the precision of exposure-outcome associations. Second, we employed a data-driven feature selection and ranking approach with an emphasis on explainability. Our approach enables transparent identification of key predictors and quantification of their contributions to biological age prediction. This balance between predictive performance and interpretability enhances the translational potential of wearable-based digital biomarkers to future research.

Nonetheless, this study has limitations. First, UK Biobank participants are generally healthier and predominantly of European ancestry, which may limit the generalizability of our findings. Replication in more diverse population across geographic regions, age groups, and racial backgrounds is warranted. Second, only 45% of UK Biobank participants have linked electronic health record data, which may be incomplete due to delayed or selective billing practices. However, previous studies have demonstrated that UK Biobank general practitioner records yield reasonably accurate laboratory values, supporting their utility in large-scale epidemiological research^32^. Third, the UK Biobank cohort includes individuals aged 40–70 years, which may limit generalizability to younger or older populations. However, this age range marks a critical window for chronic disease onset and lifestyle transitions driven by biological and environmental factors, emphasizing the importance of early risk identification. Next, to assess systemic inflammation, we used SII. While more detailed inflammation markers (e.g., CRP) were not available for a large subset of individuals, SII can be derived from routine blood tests, provides broad inflammatory insights, and is shown to predict several aging-related outcomes, including cardiovascular disease, cancer, and overall mortality in large-scale studies. In addition, cosinor modeling relies on assumptions of stationarity and sinusoidality, which may not fully capture irregular or non-sinusoidal activity patterns. To address this, we incorporated a rhythm regularity screening step as part of the inclusion criteria and excluded individuals with extremely irregular or fragmented rhythms to ensure valid estimation of rest-activity rhythm metrics. This screening was intended to remove recordings in which the observed irregularity could not be confidently attributed to rest-activity organization, as it may reflect unmeasured exogenous disruptions or device-related artifacts not captured by available metadata. In addition, the number of deaths was small, particularly in the sex-stratified analyses, which may have limited statistical power for detecting indirect effects in the mediation analyses.

Our analysis is also retrospective and observational, which limits causal inference; thus, all reported associations should be interpreted as correlational and reflective of potential pathways rather than causal relationships. Moreover, actigraphy-derived rhythmicity reflects behavioral rest-activity patterns rather than endogenous circadian regulation and may be influenced by external social or environmental factors. To mitigate this, we included employment status and shift-work history as covariates and conducted sensitivity analyses excluding individuals with shift work exposure or sleep disturbance and found the associations remained consistent. Residual collinearity among features may still influence SHAP interpretations, even after we applied the MRMR feature selection procedure, which takes collinearity into account. Finally, current wearable devices rely on a 7-day monitoring window, which provides limited biological specificity and constrains inferences about longer-term rhythm stability or biological aging trajectories. Future work incorporating extended longitudinal monitoring would strengthen the evaluation of rhythm stability and further validate the proposed methodology.

In conclusion, this study identifies wearable-derived behavioral features associated with biological aging, demonstrates sex-specific links to systemic inflammation, and suggests systemic inflammation as a potential pathway through which disrupted rest-activity rhythms are associated with mortality. Continued work using longitudinal and experimental designs will be essential for establishing causal pathways and evaluating the effectiveness of personalized digital interventions aimed at modulating inflammation and improving healthspan.

## Methods

### Data sources and study population

**Fig. 3:**
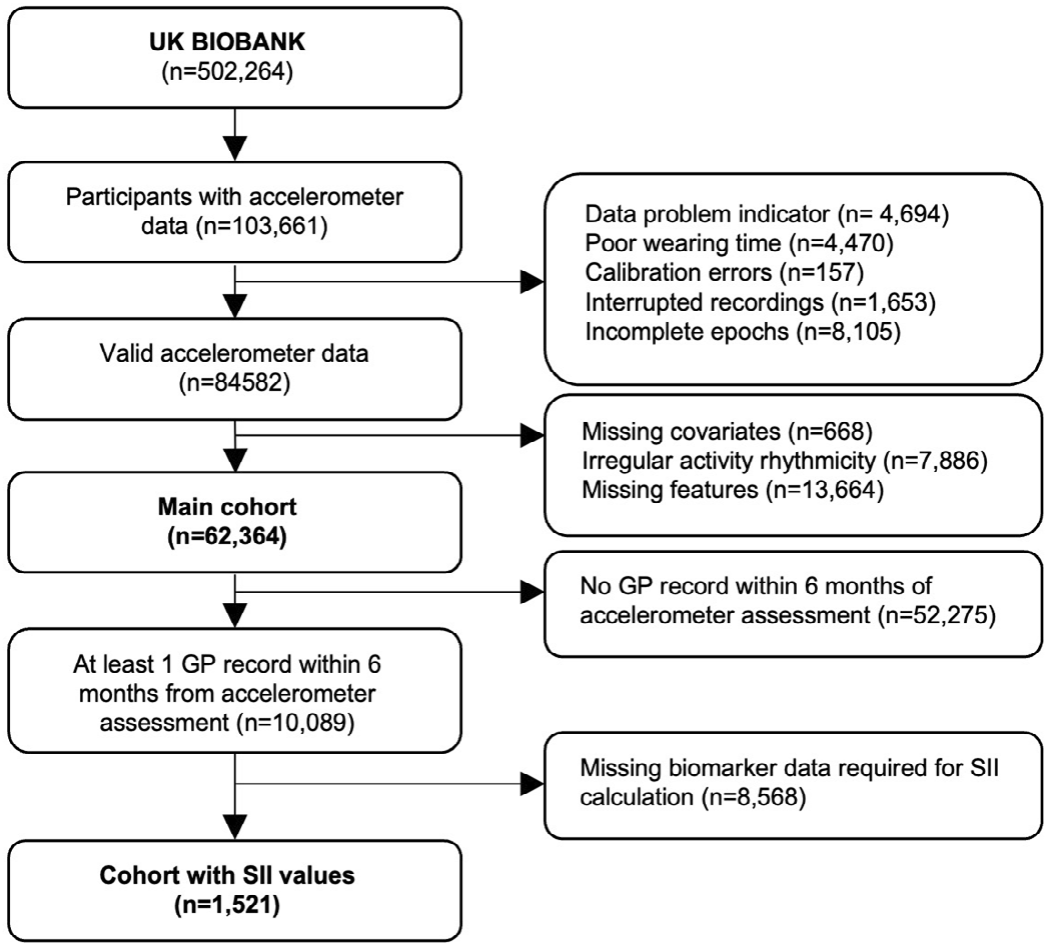
Flowchart of study cohort inclusion. GP UK Biobank primary care electronic health records, SII systemic inflammation index.

Figure 3 describes the study cohort inclusion. We utilized a prospective cohort from the UK Biobank study, a large-scale population-based study comprising over 500,000 participants aged 40–69 years at baseline assessment (2006–2010). The UK Biobank received ethical approval from the National Health Service National Research Ethics Service (16/NW/0274), and all participants provided written informed consent. For this study, we selected participants from the accelerometer assessment conducted between June 2013 and December 2015^13,33^. Data from 103,681 participants, who were equipped with an Axivity AX3 wrist-worn triaxial accelerometer (Axivity Ltd, Newcastle, UK), which recorded raw acceleration data at 100 Hz, were available at the time of analysis. After excluding individuals with data problems flagged by UK Biobank, 84,582 participants remained with valid accelerometer recordings. To construct the main analytic cohort (*n* = 62,364), we removed participants with missing data on covariates (*n* = 668), primarily in socioeconomic variables such as education (*n* = 445), employment (*n* = 82), and TDI (*n* = 79). Next, to ensure valid cosinor model fits, individuals exhibiting extremely irregular or fragmented activity patterns were excluded (*n* = 7886). Rest-activity rhythm regularity and fragmentation were quantified using interdaily stability (IS) and intradaily variability (IV), excluding participants with IS<5th percentile or IV>95th percentile of the sample distribution. From the remaining data, 43 wearable-derived features were computed (see Wearable Accelerometer Data Processing section). Participants with complete feature sets were retained, yielding 62,364 individuals in the final analytic cohort. This cohort was used for explaining biological aging via wearable-derived digital biomarkers using machine learning and explainable AI.

Among them, 10,089 had at least one general practitioner (GP) record within 6 months of the accelerometer assessment. We tested different time windows of 3 months and 12 months to assess the robustness of the inflammation relationship across varying time periods. The final choice of a 6-month window was made to balance sample size with maintaining a proximity to the accelerometer measurement, ensuring that the inflammation marker levels were reflective of more immediate physiological states. Finally, a subcohort of 1521 individuals had complete biomarker data for systemic immune-inflammation index (SII). This subcohort was used to evaluate associations between wearable features and systemic inflammation, to examine the joint effect of activity rhythms and physical activity on inflammatory markers, and to perform mediation analysis. This research was conducted using the UK Biobank Resource under Application Number 102250. All methods were performed in accordance with the Declaration of Helsinki.

### Explaining biological aging via wearable-derived digital biomarkers

#### Identifying feature set

First, we created a comprehensive list of features from wearable devices based on literature review. We searched PubMed for journal articles published between January 1, 2007 (the advent of iPhone) and June 1, 2024, using the combination of terms (“circadian rhythm” or “rest-activity” or “rest activity” or “circadian rest activity rhythm”) AND (“actigraph” or “accelerometer” or “wearable” or “smartphone”) AND (“prospective” or “cohort” or “longitudinal”), without language restrictions. We excluded studies that only included participants younger than 45 years old, study protocols, or pre-prints, resulting in 42 studies. Our final list included 43 features categorized into one of four domains: rest-activity rhythm, physical activity, sleep, and data-adaptive approach. A detailed definition of each feature is provided in Supplementary Table 1.

#### Wearable accelerometer data processing

Next, we expressed the movement-intensity signal from the wearable accelerometer using the minute-level Euclidean Norm Minus One (ENMO) metric. One gravitational unit (1 g) was subtracted from the vector magnitude of acceleration across three axes, with all negative values set to zero. We applied data quality metrics developed by the UK Biobank accelerometer working group to exclude participants with unreliable accelerometry data based on the following conditions: 1) accelerometer data flagged by UKB-provided data problem indicator (Field ID: 90002) as either “unreliable due to unexpectedly small size” or “unreliable due to unexpectedly large size”; 2) insufficient wear time (<72 h or no wear data in each 1-h period of the 24-h cycle; Field ID: 90015); 3) unreliable or poorly calibrated data (Field IDs: 90016 & 90017); and 4) accelerometry data with nonzero counts of interrupted recording periods (Field ID: 90180). We then extracted the 43 features from the preprocessed wearable data. Rest-activity rhythm features were obtained using an extended cosinor model via the cosinor2 package and nonparametric features using the nparACT package. The models were fit on both the full time series and individual days. The former captured overall rhythmicity, while the latter quantified day-to-day variability of features using the coefficient of variation^34^. This distinction is essential for distinguishing between consistently disrupted rhythms and a robust but misaligned rhythm, both of which may present with low amplitude or reduced goodness of fit from an extended cosinor model. Physical activity features, including MVPA and sedentary behavior duration, were computed based on validated cut-offs, specifically >100 mg/min for MVPA and <40 mg/min for sedentary behavior^13,34^. Sleep features, such as sleep duration, sleep efficiency, and sleep onset time, were computed using GGIR package by Jones et al.^30^.

For data-adaptive approaches, we applied functional principal component analysis (fPCA) and detrended fluctuation analysis (DFA) (Supplementary Table 1). Applying fPCA to the averaged 24-h accelerometer data, we extracted the first four principal components, which capture the dominant patterns of daily activity rhythmicity^35^. DFA characterizes the temporal complexity of activity fluctuations; healthy physiological signals exhibit fractal fluctuations with self-similar structures, while reduced fractal complexity has been associated with compromised health and aging-related decline^36^. We computed DFA scaling exponents over short (<2 h) and long (>2 h) time scales.

#### Machine learning model development

For feature selection, we employed the minimum redundancy-maximum relevance (MRMR) method^37^, a filtering-based feature selection method which reduces feature redundancy while preserving the most relevant ones for model performance. MRMR ranks features by maximizing their mutual information with the outcome (maximum relevance) while minimizing inter-feature correlation (minimum redundancy), an approach particularly advantageous for high-dimensional wearable-derived datasets where behavioral and sensor-derived features may be intercorrelated. MRMR is also computationally efficient compared with wrapper method (e.g., recursive feature elimination) in large-scale datasets. Moreover, it is model-agnostic and can capture nonlinear, non-monotonic associations, making it more suitable than embedded methods (e.g., LASSO or Elastic Net) that rely on linearity assumptions and shrinkage-based penalization. The effectiveness of MRMR has been demonstrated in various studies that utilized accelerometer and sensor data from digital devices, including activity pattern recognition^38^, Parkinson’s disease detection^39^, and depression monitoring^40^. In our study, we retained features with a pairwise Spearman correlation coefficient >0.7, resulting in 25 input features for the classification of accelerated vs decelerated biological aging. Features are standardized to have a zero mean and a unit variance.

The classification task was carried out using five machine learning models: logistic regression, random forest, support vector classifier, decision tree, and extreme gradient boosting (XGBoost). Accelerated aging was quantified using CosinorAge, a wearable-derived biological age metric that we previously developed^13^. CosinorAge is a wearable-derived digital biomarker of aging that estimates an individual’s biological age based on accelerometer-measured rest-activity rhythm patterns^13^. It was designed to estimate individual 5-year mortality risk from rest-activity rhythm metrics extracted from wearable accelerometry using Gompertz proportional hazard models. Sex-specific model weights were applied to estimate wearable-derived biological age for each individual, reflecting their physiological aging status relative to chronological age.

To ensure unbiased evaluation, the dataset was randomly split into 80% for model training (65% for training set and 15% for validation set) and 20% for testing set. During model development, the model was trained exclusively on the training set, while the validation set was used for hyperparameter optimization. After identifying the optimal hyperparameters, the final model was retrained on the entire 80% training data (combining the training and validation sets) and then evaluated on the unseen test set. The performance of each model was evaluated using the area under the receiver operating characteristic curve (AUC), accuracy, and the Matthews correlation coefficient (MCC). We employed a grid search to identify the optimal parameters, with the final hyperparameters for each model summarized in Supplementary Table 2.

#### Explainable AI

To interpret feature contributions, we employed SHAP (SHapley Additive exPlanations) based on tailored to each model type^41,42^. SHAP is a local attribution method based on cooperative game theory, assigning a contribution value to each feature that reflects its marginal influence on a specific prediction, SHAP values quantified each feature’s marginal influence on predictions, allowing identification of the top features contributing to the model output. SHAP was employed as part of an interpretable machine learning framework designed to balance predictive accuracy and interpretability. Linear models offer direct interpretability but are limited in capturing the nonlinear and multidimensional relationships characteristic of wearable-derived behavioral data. Our approach leverages machine learning to model such complex dependencies while using SHAP as a model-agnostic tool to interpret and explain feature contributions. SHAP quantifies nonlinear and interaction effects across high-dimensional predictors, provides interpretable feature attributions at both individual and population levels, and enables consistent comparisons across different machine learning models.

### CosinorAge estimation

CosinorAge is a rest-activity rhythm-based biological aging metric derived from wrist-worn accelerometry^13^. First, raw tri-axial acceleration signals from UK Biobank and NHANES were processed into minute-level Euclidean norm minus one (ENMO) values following standardized quality control procedures. We leveraged two population-level datasets, UK Biobank (UKB) and US NHANES in the model development and validations. In UKB, 103,661 participants from the 2013 to 2015 subsample provided usable wrist-worn accelerometer data (Axivity AX3, 100 Hz). Raw acceleration was converted to ENMO values and aggregated to the minute level. Following UKB quality criteria, participants were excluded due to data issues, insufficient wear time (<72 h), poor calibration, interrupted recordings, or any missing 5-min epoch within the 24-h cycle. In NHANES, 14,693 participants from the 2011 to 2014 cycles wore a wrist-worn accelerometer (ActiGraph GT3X+, 80 Hz) for 7 days. Raw signals were processed into minute-level ENMO values using the *SummarizedActigraphy* R package. Participants were excluded for data quality problems, insufficient wear (<16 h/day or <4 days), or missing data in any 5-min epoch.

Then, we characterized the 24-h rest-activity rhythm using cosinor analysis, which examines the degree of fit between the wearable data and a superposition of cosine functions^43,44^. The cosinor model has been widely applied to analyze circadian markers based on cortisol, melatonin, and core body temperature^45,46^. A single component cosinor model is written as in Eq. (1):

1 $$Y\left(t\right)=M+A\cdot \cos \left(\frac{2\pi t}{\tau }+\,\varphi \right)+e\left(t\right)$$

where *Y(t)* refers to the accelerometry activity level at time t. *M* indicates the rhythm-adjusted average activity level, known as the Midline Estimating Statistic of Rhythm (MESOR). Amplitude (*A*) is a measure of half the extent of the variation within the cycle. Acrophase (*φ*) measures the time of the maximum activity level. Period (*τ*) is a duration of one cycle, and *e(t)* is the error term. The non-linear problem of fitting a cosine function is reduced to a problem with linear parameters by applying the least squares methods as follows, thus allowing to extract rest-activity rhythm parameters. The best fitting period of the cosinor model of 24-h was estimated using iterative cosinor procedures.

These rest-activity rhythm parameters were incorporated into a parametric proportional hazards model with a Gompertz distribution to estimate each participant’s 5-year mortality risk. Rest-activity rhythm parameters (MESOR, amplitude, acrophase) together with chronological age were included as predictors. A second Gompertz model was fitted using chronological age alone. CosinorAge was defined by equating the predicted mortality risks from the rest-activity rhythm-based model and the age-only model and solving analytically for age. This yielded a biological age measure derived from rest-activity rhythm. Step-by-step derivation can be found in Supplementary Methods of Shim et al.^13^.

Biological age acceleration was defined as difference between the estimated biological age and actual chronological age of an individual. Positive values indicate accelerated biological aging and values at or below zero reflect decelerated aging. This framework parallels established biological aging measures such as PhenoAge and Klemera-Doubal Age.

Strengths of the approach include its development and external validation in two large population-based cohorts and its robust prediction of mortality, disease incidence, and functional decline. Limitations include reliance on a single 7-day accelerometry window. It is also worth noting that UKB participants tend to be healthier and predominantly of White race/ethnicity compared to the general UK population. Lastly, CosinorAge was developed among middle-aged and older adults, and its applicability to younger age groups may not be fully generalizable.

### Ascertainment of outcome

To examine the relationship between wearable-derived features relevant to biological age prediction and proximal inflammation, we utilized UK Biobank primary care electronic health records (GP data) to extract inflammation marker levels measured near the time of accelerometer assessment. GP data offer a time-stamped, longitudinal summary of key clinical information-including disease progression, diagnostic findings, treatments, and prescribed medications providing a structured basis for evaluating a patient’s current health status at the time of physiological monitoring^31^. Approximately 45% of the UK Biobank cohort contributes to the GP data, and previous studies have demonstrated the feasibility of utilizing GP data to identify clinical biomarkers, including inflammatory markers^31^.

In a subset of the cohort with complete laboratory tests, we assessed the level of systemic immune-inflammatory index (SII) within six months of the wearable data collection. SII is calculated by: SII = Platelet count × Neutrophil count/Lymphocyte count. We searched the linked electronic health records using pre-identified “Read v2” and “Read v3” codes (Supplementary Table 3).

For mediation analysis, we considered all-cause death as an outcome. Mortality records were linked to the UKB dataset from NHS Digital (England and Wales) and the Information and Statistics Division (Scotland), and the censoring date is set to 2022-11-30. Participants were followed from the start date of accelerometer wear to the date of death or to the censoring date for survivors, with follow-up time expressed in years.

### Covariates

To evaluate the associations between wearable-derived features and inflammation markers, we selected covariates known from prior research^47,48^: Age, sex (female vs male), ethnicity/race (white vs non-white), and Townsend Deprivation Index (TDI) were collected during the baseline assessment. The TDI quantifies community-level socioeconomic deprivation accounting for four census-based indicators: unemployment, non-home ownership, household overcrowding, and non-car ownership^49^. In the UKB, each participant’s TDI score was derived from their residential postcode, mapped to the deprivation score from the latest national census^50^. Employment status (paid employed vs unemployed) and shift work status (yes vs no) were derived from baseline self-reported questionnaires. Body mass index (BMI) was categorized to align with the World Health Organization^51^ and previous research standards^47^ as normal or underweight (≤25 kg/m²), overweight (25–29.9 kg/m^2^), and obese (≥30 kg/m^2^). Smoking status (never vs previous vs current) and alcohol consumption (not current drinker, non-regular drinker (<3 times/week), and regular drinker (≥3 times/week) were determined from the baseline touch-screen questionaries during the assessment visit^52^. Specifically, smoking status was assessed using questions on current tobacco smoking (“Do you smoke tobacco now?”: “yes, on most of all days”, “only occasionally”, “no”, and “prefer not to answer”) and past tobacco smoking (“In the past, how often have you smoked tobacco?”: “Smoked on most of all days”, “smoked occasionally”, “just tried once or twice”, “I have never smoked”, “prefer not to answer”). Alcohol consumption was assessed with the question “How often do you drink alcohol?” with response options of “prefer not to answer”, “never”, “special occasions only”, “one to three times a month”, “once or twice a week”, “three or four times a week”, “daily or almost daily”. Comorbidities were identified a composite measure across self-reported touchscreen questionnaires, self-reported interviews, and linked hospital admission and death records (using ICD-10 codes). We included six common chronic conditions: hypertension, type 2 diabetes, cardiovascular disease, cancer, neurodegenerative disease, and chronic respiratory diseases (Supplementary Table 5).

### Sleep disturbance

We identified sleep disturbance using two complementary approaches to ensure accurate assessment^53,54^. First, insomnia symptoms were identified from self-reported difficulty falling asleep or maintaining sleep (“Do you have trouble falling asleep at night or do you wake up in the middle of the night?”), with participants responding “Usually” classified as experiencing sleep disturbance. Second, an expanded definition additionally incorporated habitual snoring (“Does your partner or a close relative or friend complain about your snoring?”) and excessive daytime sleepiness (“How likely are you to doze off or fall asleep during the daytime when you don’t mean to? (e.g. when working, reading or driving)”). Participants reporting snoring complaints or frequent unintentional daytime dozing (“Often” or “All of the time”) were categorized as having sleep disturbance. Participants were classified as experiencing sleep disturbance if they reported one or more symptoms of insomnia, snoring, or daytime dozing.

### Statistical analysis

The baseline demographics and wearable features were summarized as median and interquartile range (IQR) for continuous variables, and count (N) and percentage (%) for categorical variables. The *p*-values in descriptive statistics are computed using Wilcoxon rank-sum test for continuous variables and chi-square test for categorical variables.

To evaluate the associations between wearable features and inflammation markers, we employed multivariable generalized linear models in a subset of participants with available systemic immune-inflammation index data (*n* = 1521). For the main effect, the models are adjusted for pre-specified covariates (see Covariates subsection), including age, sex, ethnicity/race, TDI, BMI, employment status, shift work status, smoking status, alcohol consumption, sleep duration, and comorbidities such as hypertension, Type 2 diabetes, cardiovascular disease, cancer, neurodegenerative disease, and chronic respiratory disease. For relative amplitude and interdaily stability, models were additionally adjusted for MVPA duration. For MVPA, models were additionally adjusted for interdaily stability. Interaction effect models were adjusted for age, sex, ethnicity/race, TDI, BMI, employment status, and shift work for convergence. The outcome variable (SII) was Z-scored prior to analysis, and regression coefficients therefore represent standardized effects per one standard deviation (1 SD) increase in SII. All statistical tests were two-sided, and a *p*-value less than 0.05 was considered as statistical significance. We included the false discovery rate procedure by Benjamini and Hochberg^55^ as sensitivity analysis to account for multiple testing.

Wearable-derived features were categorized into quartile groups for easier interpretation of the associations with inflammation markers. In statistical models, both categorical (quartile) and continuous (numeric trend) forms were examined. Specifically, *relative amplitude (quartiles)* classifies participants into four groups from high to low amplitude, whereas *relative amplitude (continuous)* represents the corresponding numeric trend across these quartiles. Likewise, *interdaily stability (quartiles)* categorizes participants from good regularity to greater irregularity, and *interdaily stability (continuous)* captures the numeric trend across the same gradient. Detailed quartile cutpoints are provided in Supplementary Table 6. For physical activity, we also categorized participants to have “sufficient MVPA” if having 150 min or more moderate-to-vigorous intensity activity per week, and “insufficient MVPA” otherwise^56^. Sleep variability was categorized as “irregular sleep” if the standard deviation of sleep duration exceeded 90 min during the monitoring period, and “regular sleep” if the standard deviation was below this threshold^57^.

For the mediation analysis, we adopted a causal mediation framework to investigate whether systemic inflammation mediates the relationship between wearable digital biomarkers and overall aging. We first assessed the associations between wearable-derived features, specifically low amplitude, insufficient MVPA, and rhythm irregularity, and all-cause mortality in full cohort (*n* = 62,364) using multivariable Cox proportional hazard models implemented in survival R package. Next, causal mediation analyses evaluated the association between wearable-derived features and all-cause mortality, with systemic inflammation as a mediator. This is a counterfactual-based natural effect models where total effect, natural direct effect, and natural indirect effect of wearable features on mortality risk are estimated on a hazard ratio scale^58–60^. The natural direct effect is the effect that an exposure has on the outcome through pathways not involving the mediator. Natural indirect effect is the effect of exposure on the outcome entirely through the mediator. We estimated hazard ratios (HRs) for the total, natural direct, and natural indirect effects for rhythm irregularity, insufficient MVPA, and low amplitude as predictors, and the 95% confidence intervals (CIs) were derived from 1000 bootstrap samples using the 2.5th and 97.5th percentiles using medflex R package^60^. The proportion mediated was calculated using natural indirect log-HR divided by total effect log-HR^58^. All mediation models were adjusted for age, TDI, BMI, employment status, and shift work. To ensure robustness of findings, several sensitivity analyses were conducted. First, we excluded participants reporting shift work or sleep disturbances (insomnia, habitual snoring, or daytime sleepiness) to evaluate whether irregular work schedules or impaired sleep patterns influenced the observed associations. Next, we stratified the study population by age (<60 years vs ≥60 years) and re-ran the analyses to assess the associations. The age cut-off at 60 years was chosen based on evidence showing an abrupt shift in immune regulation at this age. Additionally, we considered the potential impact of the transition to retirement, which is associated with significant changes in rest-activity rhythm and physical activity patterns around age 60^13^. Statistical analyses were conducted using R (version 4.2) and Python (version 3.11).

## Supplementary information

SUPPLEMENTAL MATERIAL

## Data Availability

The UK Biobank data are available for research purposes by application (http://www.ukbiobank.ac.uk). The codes that support the findings of this study are available upon request addressed to the corresponding author.

## Code availability

The UK Biobank data are available for research purposes by application (http://www.ukbiobank.ac.uk). The codes that support the findings of this study are available upon request addressed to the corresponding author.
