# Supplementary material for: From wrist data to lifespan: elucidating inflammation-driven biological aging via activity rhythms captured by wearable devices
Source: NPJ Aging. 2026 Feb 26;12(1):49. doi: 10.1038/s41514-026-00349-x (PMC13046744; doi:10.1038/s41514-026-00349-x)
Supplement: Supplementary file 1 — SUPPLEMENTAL MATERIAL [file 41514_2026_349_MOESM1_ESM.pdf]

SUPPLEMENTAL MATERIAL

Title: From Wrist Data to Lifespan: Elucidating Inflammation-Driven Biological Aging via Activity Rhythms Captured by Wearable Devices

Authors: Jinjoo Shim[1,2*], Faraz Bishehsari[3,4], Mahboobeh Mahdavinia[5], Jamie M. Zeitzer[6], Elgar Fleisch[1,7], Filipe Barata[1]

Affiliations:

[1]Centre for Digital Health Interventions, ETH Zurich, Zurich, Switzerland;

[2]Department of Biostatistics, Harvard University, Boston, MA 02115, USA;

[3]Gastroenterology Research Center (GRC), Department of Internal Medicine, University of Texas Health Science Center at Houston, TX 77030, USA;

[4]MD Anderson Cancer Center-UTHealth Houston Graduate School of Biomedical Sciences, Houston, TX 77030, USA;

[5]Division of Allergy and Immunology, Department of Internal Medicine, University of Texas Health Science Center at Houston, Houston, TX 77030, USA;

[6]Department of Psychiatry and Behavioral Sciences, Stanford University, Stanford, CA 94305, USA;

[7]Centre for Digital Health Interventions, University of St. Gallen, St. Gallen, Switzerland

Corresponding author: Jinjoo Shim (jinjooshim@hsph.harvard.edu)

Supplementary Table 1. Definition of Wearable-derived Accelerometer-measured Features

Supplementary Table 2. Final Hyperparameters of ML Models

Supplementary Table 3. Read Codes Used for Identifying Biomarkers from UK Biobank Primary Care Electronic Health Records

Supplementary Table 4. Performance Metric Summary on Test Dataset

Supplementary Table 5. Codes to Identify Comorbidities in UK Biobank

Supplementary Table 6. Quartile Cutpoints for Relative Amplitude and Interdaily Stability

Supplementary Table 7. Sensitivity Analysis of Associations Between Wearable Features and Systemic Inflammation Excluding Shift Workers

Supplementary Table 8. Sensitivity Analysis of Associations Between Wearable Features and Systemic Inflammation Excluding Participants with Sleep Disturbance

Supplementary Table 9. Wearable Features and All-cause Mortality in Full Cohort (n=62,364)

Supplementary Table 1. Definition of Wearable-derived Accelerometer-measured Features

Feature	Domain	Definition
**Cosinor amplitude**	Rest-Activity Rhythm	A measure of half the extent of predictable variation within a cycle
**MESOR**		Midline Estimating Statistic Of Rhythm, a rhythm-adjusted mean
**Up-mesor**		Time of day of switch from low to high activity
**Down-mesor**		Time of day of switch from high to low activity
**Acrophase**		Time of peak activity in fitted cosinor model
**Circadian quotient**		Cosinor amplitude divided by MESOR
**Pseudo F**		Model goodness-of-fit measure; robustness of rest-activity rhythm
**Intradaily variability**		Measure of rhythm fragmentation
**Interdaily stability**		Day-to-day rhythm regularity
**Autocorrelation coefficient**		Similarity between activity patterns over a 24-h period
**Relative amplitude**		Difference between peak and trough activity normalized
**M10**		Mean activity during the 10 most active consecutive hours in a 24-h day
**L5**		Mean activity during the 5 least active consecutive hours in a 24-h day
**Intensity gradient**	Physical Activity	Intensity distribution of activity during the day
**Sedentary behavior**		Activity duration < 40 mg/minute
**Light physical activity**		Activity duration ≥ 40 mg/minute and ≤ 100 mg/minute
**Moderate-to-vigorous physical activity (MVPA)**		Activity duration > 100 mg/minute
**Total activity**		Daily total physical activity (mg)
**Physical activity energy expenditure**		Estimated daily physical activity energy expenditure
**Sleep efficiency**	Sleep	Sleep duration divided by length of sleep period time windows
**Total sleep time**		Daily sleep duration
**Sleep onset**		Time of sleep onset
**Sleep midpoint**		Midpoint between sleep onset and wake time
**Functional principal components**	Data-Adaptive	First four principal components derived from functional data analysis.
**Detrended fluctuation analysis**		Fractal scaling exponent using shorter-time scale (< 2 hours) and longer-time scale (> 2 hours).
**Cosinor Amplitude (variability)**	Rest-Activity Rhythm	Day-to-day variation in cosinor amplitude
**MESOR (variability)**		Day-to-day variation in MESOR
**Up-mesor (variability)**		Variability in up-mesor timing
**Down-mesor (variability)**		Variability in down-mesor timing
**Acrophase (variability)**		Day-to-day variability in peak timing
**Circadian quotient (variability)**		Day-to-day variation in circadian quotient
**Relative amplitude (variability)**		Daily variability in relative amplitude
**M10 (variability)**		Day-to-day variability in M10
**L5 (variability)**		Day-to-day variability in L5
**Sedentary behavior (variability)**	Physical Activity	Daily variation in sedentary time
**Light physical activity(variability)**		Daily variation in light-intensity activity
**MVPA (variability)**		Daily variation in moderate-to-vigorous physical activity
**Total activity (variability)**		Daily variation in total activity
**Total sleep time (variability)**	Sleep	Day-to-day variation in total sleep duration

Rest-activity rhythm: This domain quantifies the temporal organization, phase, and stability of behavioral rest-activity rhythms. Metrics include both parametric cosinor analysis features (cosinor amplitude, MESOR, up-mesor, down-mesor, acrophase, circadian quotient, pseudo F) and nonparametric rhythm analysis features (intradaily variability, interdaily stability, autocorrelation coefficient, relative amplitude, M10, L5). Cosinor analysis models the activity signal as a sinusoidal function estimating the MESOR (a rhythm-adjusted mean), amplitude (a measure of half the extent of predictable variation within a cycle), and acrophase (timing of peak activity). In contrast, nonparametric indices describe day-to-day regularity (IS), within-day fragmentation (IV), and relative amplitude between active and rest periods (RA) without assuming a fixed waveform. These measures complement one another by capturing both the form and robustness of the 24-hour rest-activity rhythm. Higher RA and stronger IS indicate a well-synchronized and consolidated rhythm, whereas reduced amplitude or IS reflects rest-activity rhythm disruption, behavioral irregularity, or fragmentation of activity patterns. In addition, for rest-activity rhythm, physical activity, and sleep, we further included daily variability metrics to characterize intraindividual fluctuations over monitoring period, thereby capturing day-to-day fluctuation and instability beyond average-level estimates.

Physical activity: This domain quantifies the magnitude and intensity of movement independent of activity timing. Metrics include light physical activity, moderate-to-vigorous physical activity (MVPA), sedentary time, intensity gradient, and total activity energy expenditure. Activity levels were determined using predefined acceleration thresholds (in milligravity, mg): <40 mg/min for sedentary behavior, 40-100 mg/min for light activity, and >100 mg/min for MVPA. These measures characterize behavioral output rather than rhythmic organization, providing indices of overall activity load. In contrast to rest-activity rhythm features that capture when and how regularly activity occurs, physical activity features quantify how much and how intensely movement is performed.

Sleep: This domain comprises features describing the duration, timing, and efficiency of nocturnal rest derived from low-activity periods detected by accelerometry. Measures such as total sleep time, sleep efficiency, and sleep onset quantify sleep consolidation and timing. These parameters provide complementary information to rest-activity rhythm and physical activity domains by reflecting behavioral expression of the sleep homeostat and recovery-related processes.

Data-adaptive approach: This domain encompasses data-driven, algorithmically derived features that capture latent temporal structures beyond predefined behavioral definitions. Examples include functional principal component analysis (fPCA) and detrended fluctuation analysis (DFA). These adaptive metrics characterize individual-specific temporal dynamics not captured by traditional parametric or threshold-based approaches.

- **Functional Principal Data Analysis (fPCA):** To characterize higher-order latent patterns in daily activity rhythm profiles, we applied functional principal component analysis (fPCA)[1]. Wearable-derived 24-hour activity rhythm was treated as functional data characterized with a continuous and smooth temporal process[2]. For each participant, minute-level average wearable accelerometer data was obtained across all available days, yielding 1440 observations per participant. We then projected these trajectories onto a nine-term Fourier basis, consistent with prior studies that effectively captures diurnal periodicity with minimal overfitting[2,3]. The resulting smoothed functions

were subjected to fPCA, which decomposes the covariance matrix into an orthonormal basis of eigenfunctions that capture principal components modes of variation. We retained the first four functional principal components (fPCs), which jointly explained 81% of the total variance. Each participant was represented by a vector of four orthogonal functional principal component (fPC) scores. These scores capture distinct and interpretable dimensions of behavioral rhythm at the population level (**Supplementary Figure 1**). Specifically, fPC1 reflects the high vs. low overall amplitude; fPC2 represents the later vs. early rise times; fPC3 captures morning vs. evening peaks; and fPC4 distinguishes unimodal from bimodal activity patterns. Consistent with findings from previous studies[4], these behavioral rest-activity rhythm characteristics present volume, timing, structure, and regularity.

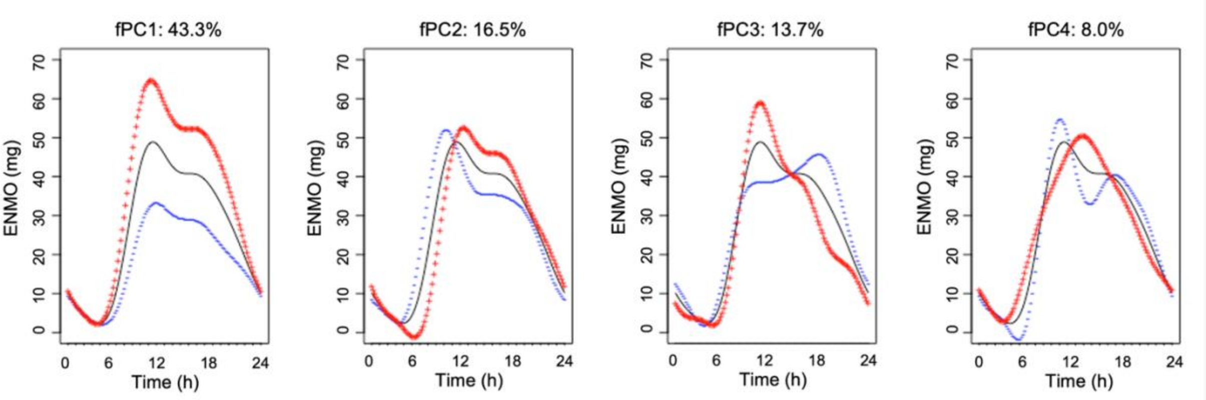

Supplementary Figure 1. Functional principal component analysis (fPCA) of 24-hour wearable-derived activity rhythm. Each panel shows the mean daily activity pattern (black) and variations corresponding to positive (“+”, red) and negative (“–”, blue) directions of the first four fPCs. fPC1 reflects high vs. low overall amplitude, fPC2 represents later vs. early rise times, fPC3 captures morning vs. evening peaks, and fPC4 differentiates unimodal from bimodal activity patterns. The percentage in each panel indicates the variance explained by each component.

- **Detrended Fluctuation Analysis (DFA):** We estimated the fractal scaling exponent (α) of activity fluctuations using detrended fluctuation analysis (DFA), a method applied to wearable accelerometry data and associated with health outcomes such as cognitive function[5] and frailty and mortality[6]. The exponent was calculated separately for shorter (< 2 h; α_1) and longer (> 2 h; α_2) time scales to differentiate short-term behavioral organization from long-range autocorrelation[6]. Higher α values denote stronger temporal correlations and more stable activity dynamics, whereas values near 0.5 indicate randomness.

Supplementary Table 2. Final Hyperparameters of ML Models

Model	Hyperparameters
**XGBoost**	{‘colsample bytree’: 0.7, ‘gamma’: 0, ‘learning rate’: 0.1, ‘max depth’: 10, ‘min child weight’: 10, ‘subsample’: 1.0}
**LR**	{‘penalty’: ‘l1’}
**SVC**	{‘C’: 10, ‘gamma’: 0.01}
**DT**	{‘criterion’: ‘gini’, ‘max depth’: 7, ‘min samples leaf’: 4, ‘min samples split’: 10}
**RF**	{‘criterion’: ‘entropy’, ‘min samples split’: 5, ‘n estimators’: 500}

XGBoost, extreme gradient boosting; LR, logistic regression; SVC, support vector classifier; DT, decision tree; RF, random forest.

Supplementary Table 3. Read Codes Used for Identifying Biomarkers from UK Biobank Primary Care Electronic Health Records

Biomarker	Read Codes
**Platelet**	42P..00, 42P1.00, 42PZ.00, 42P2.00, 42P3.00, 42P4.00, 42P.., 42P1., 42PZ., XE24o, 42P3.
**Neutrophils**	42J..00, 42JZ.00, 42J..11, 42J2.00, 42J1.00, 42J3.00, 42J4.00, 42J.., 42JZ., 42J2., 42J1., 42J3., 42J4.
**Lymphocytes**	42M..00, 42MZ.00, 42M1.00, 42M4.00, 42M.., 42MZ., 42M1.

Supplementary Table 4. Performance Metric Summary on Test Dataset

Model	AUC	Sensitivity	Specificity	Accuracy	MCC	PPV	NPV	F1-score
**XGBoost**	0.971	0.889	0.915	0.903	0.805	0.905	0.9	0.897
**LR**	0.943	0.856	0.871	0.864	0.728	0.859	0.869	0.858
**SVC**	0.965	0.882	0.901	0.892	0.784	0.891	0.893	0.887
**DT**	0.947	0.848	0.895	0.872	0.744	0.881	0.865	0.864
**RF**	0.964	0.872	0.909	0.891	0.782	0.898	0.885	0.885

AUC, area under the receiver operating characteristic curve; MCC, Matthews correlation coefficient; PPV, positive predictive value; NPV, negative predictive value; XGBoost, extreme gradient boosting; LR, logistic regression; SVC, support vector classifier; DT, decision tree; RF, random forest.

Supplementary Table 5. Codes to Identify Comorbidities in UK Biobank

Disease Type	Self-reported: touchscreen questionnaire	Self-reported: interview	Hospital records, Death records (ICD-10)
Hypertension	6150 (4)	20002 (1065,1072, 1073)	I10-I13, I15
Diabetes	2443	20002 (1220)	E10-E14
Cardiovascular	6150 (1,2,3)	20002 (1066, 1074, 1075, 1076, 1081, 1086, 1491, 1583)	I20-I25, I46, I50, I60-I64
Cancer	2453	20001	C00-C97
Neurodegenerative	-	20002 (1259,1262,1263)	F01-F03, G122, G20, G21, G231-G233, G238, G239, G30, G31
Respiratory	6152 (6)	20002 (1112,1113,1472)	J40-J44, J47

Supplementary Table 6. Quartile Cutpoints for Relative Amplitude and Interdaily Stability

Relative Amplitude		Interdaily Stability	
Q4 (High Amplitude)	> 0.913	Q4 (Good Regularity)	> 0.379
Q3	0.894-0.913	Q3	0.337-0.379
Q2	0.869-0.894	Q2	0.299-0.337
Q1 (Low Amplitude)	< 0.869	Q1 (Irregularity)	< 0.299

Supplementary Table 7. Sensitivity Analysis of Associations Between Wearable Features and Systemic Inflammation Excluding Shift Workers

Main Effects		
**Feature**	**Beta (95% CI)**	**p-value**
**Relative Amplitude (Continuous)**	0.10 (0.04, 0.15)	0.0005*+
**Relative Amplitude (Quartiles)**		
Q4 (High Amplitude)	Reference	-
Q3	0.02 (-0.14, 0.18)	0.82
Q2	0.16 (-0.01, 0.33)	0.06
Q1 (Low Amplitude)	0.27 (0.10, 0.44)	0.0021*+
**Interdaily Stability (Continuous)**	0.05 (-0.0002, 0.11)	0.051
**Interdaily Stability (Quartiles)**		
Q4 (Good Regularity)	Reference	-
Q3	0.15 (0.001, 0.30)	0.048*
Q2	0.18 (0.03, 0.34)	0.023*
Q1 (Irregularity)	0.16 (-0.02, 0.32)	0.078
**MVPA (Categorical)**		
Male: Insufficient vs. Sufficient	0.33 (0.02, 0.630)	0.037*
Female: Insufficient vs. Sufficient	-0.001 (-0.10, 0.10)	0.983
**Interaction Effects**		
**Feature**	**Beta (95% CI)**	**p-value**
**Amplitude & Regularity**		
High Amplitude & Good regularity	Reference	-
High Amplitude & Irregularity	0.29 (-0.20, 0.79)	0.243
Low Amplitude & Good regularity	0.02 (-0.44, 0.48)	0.935
Low Amplitude & Irregularity	0.35 (-0.01, 0.72)	0.061
**MVPA & Regularity**		
Sufficient MVPA & Good regularity	Reference	-
Sufficient MVPA & Irregularity	-0.007 (-0.28, 0.27)	0.960
Insufficient MVPA & Good regularity	0.05 (-0.20, 0.30)	0.677
Insufficient MVPA & Irregularity	0.25 (0.02, 0.48)	0.037*
**Amplitude & MVPA**		
High Amplitude & Sufficient MVPA	Reference	-
High Amplitude & Insufficient MVPA	0.24 (-0.16, 0.63)	0.238
Low Amplitude & Sufficient MVPA	-0.03 (-0.38, 0.32)	0.856
Low Amplitude & Insufficient MVPA	0.31 (0.07, 0.55)	0.013*

Supplementary Table 8. Sensitivity Analysis of Associations Between Wearable Features and Systemic Inflammation Excluding Participants with Sleep Disturbance

**Main Effects**		
**Feature**	**Beta (95% CI)**	**p-value**
**Relative Amplitude (Continuous)**	0.11 (0.05, 0.16)	0.0002*+
**Relative Amplitude (Quartiles)**		
Q4 (High Amplitude)	Reference	-
Q3	0.02 (-0.15, 0.19)	0.82
Q2	0.17 (0.01, 0.34)	0.043*
Q1 (Low Amplitude)	0.29 (0.12, 0.47)	0.0008*+
**Interdaily Stability (Continuous)**	0.06 (0.004, 0.11)	0.036*
**Interdaily Stability (Quartiles)**		
Q4 (Good Regularity)	Reference	-
Q3	0.13 (-0.03, 0.28)	0.102
Q2	0.15 (-0.02, 0.30)	0.078
Q1 (Irregularity)	0.18 (0.01, 0.36)	0.038*
**MVPA (Categorical)**		
Male: Insufficient vs. Sufficient	0.32 (-0.01, 0.64)	0.054
Female: Insufficient vs. Sufficient	-0.0001 (-0.1, 0.1)	0.998

**Interaction Effects**		
**Feature**	**Beta (95% CI)**	**p-value**
**Amplitude & Regularity**		
High Amplitude & Good regularity	Reference	-
High Amplitude & Irregularity	0.22 (-0.31, 0.74)	0.417
Low Amplitude & Good regularity	0.06 (-0.44, 0.56)	0.810
Low Amplitude & Irregularity	0.41 (0.01, 0.81)	0.045*
**MVPA & Regularity**		
Sufficient MVPA & Good regularity	Reference	-
Sufficient MVPA & Irregularity	-0.019 (-0.26, 0.22)	0.875
Insufficient MVPA & Good regularity	0.05 (-0.18, 0.27)	0.669
Insufficient MVPA & Irregularity	0.20 (-0.02, 0.41)	0.074
**Amplitude & MVPA**		
High Amplitude & Sufficient MVPA	Reference	-
High Amplitude & Insufficient MVPA	0.14 (-0.20, 0.48)	0.423
Low Amplitude & Sufficient MVPA	-0.04 (-0.35, 0.27)	0.803
Low Amplitude & Insufficient MVPA	0.26 (0.04, 0.48)	0.020*

Supplementary Table 9. Wearable Features and All-cause Mortality in Full Cohort (n=62,364)

	Male		Female	
	N_{event}/N_{total}	HR (95% CI)	N_{event}/N_{total}	HR (95% CI)
Low Amplitude	1263/24948	1.62 (1.29, 2.02)	1014/37416	1.57 (1.28, 1.93)
Insufficient MVPA	1263/24948	1.22 (1.09, 1.37)	1014/37416	1.11 (0.97, 1.26)
Rhythm Irregularity	1263/24948	1.03 (0.94, 1.18)	1014/37416	0.94 (0.82, 1.06)

Cox proportional hazard models are adjusted for age ethnicity/race, TDI, BMI, employment status, shift work, smoking status, alcohol consumption, and comorbidities (hypertension, diabetes, cardiovascular disease, cancer, neurodegenerative disease, and chronic respiratory diseases).

References

1. Crainiceanu, C. M., Goldsmith, J., Leroux, A. & Cui, E. *Functional Data Analysis with R*. (Chapman and Hall/CRC, New York, 2024). doi:10.1201/9781003278726.
2. Zeitzer, J. M. *et al.* Daily Patterns of Accelerometer Activity Predict Changes in Sleep, Cognition, and Mortality in Older Men. *J Gerontol A Biol Sci Med Sci* **73**, 682–687 (2018).
3. Shi, B., Wei, P. & Huang, X. Functional principal component based landmark analysis for the effects of longitudinal cholesterol profiles on the risk of coronary heart disease. *Stat Med* **40**, 650–667 (2021).
4. Stein, M. J. *et al.* Association between circadian physical activity patterns and mortality in the UK Biobank. *International Journal of Behavioral Nutrition and Physical Activity* **20**, 102 (2023).
5. Blodgett, J. M., Ahmadi, M., Stamatakis, E., Rockwood, K. & Hamer, M. Fractal complexity of daily physical activity and cognitive function in a midlife cohort. *Sci Rep* **13**, 20340 (2023).
6. Li, P. *et al.* More random motor activity fluctuations predict incident frailty, disability, and mortality. *Science Translational Medicine* **11**, eaax1977 (2019).
